# Projection-Based Neighborhood Non-Negative Matrix Factorization for lncRNA-Protein Interaction Prediction

**DOI:** 10.3389/fgene.2019.01148

**Published:** 2019-11-20

**Authors:** Yingjun Ma, Tingting He, Xingpeng Jiang

**Affiliations:** ^1^School of Mathematics & Statistics, Central China Normal University, Wuhan, China; ^2^Hubei Provincial Key Laboratory of Artificial Intelligence and Smart Learning, Central China Normal University, Wuhan, China; ^3^School of Computer, Central China Normal University, Wuhan, China

**Keywords:** lncRNA-protein interaction, feature projection, neighborhood completion, graph non-negative matrix factorization, kernel neighborhood similarity

## Abstract

Many long ncRNAs (lncRNA) make their effort by interacting with the corresponding RNA-binding proteins, and identifying the interactions between lncRNAs and proteins is important to understand the functions of lncRNA. Compared with the time-consuming and laborious experimental methods, more and more computational models are proposed to predict lncRNA-protein interactions. However, few models can effectively utilize the biological network topology of lncRNA (protein) and combine its sequence structure features, and most models cannot effectively predict new proteins (lncRNA) that do not interact with any lncRNA (proteins). In this study, we proposed a projection-based neighborhood non-negative matrix decomposition model (PMKDN) to predict potential lncRNA-protein interactions by integrating multiple biological features of lncRNAs (proteins). First, according to lncRNA (protein) sequences and lncRNA expression profile data, we extracted multiple features of lncRNA (protein). Second, based on protein GO ontology annotation, lncRNA sequences, lncRNA(protein) feature information, and modified lncRNA-protein interaction network, we calculated multiple similarities of lncRNA (protein), and fused them to obtain a more accurate lncRNA(protein) similarity network. Finally, combining the similarity and various feature information of lncRNA (protein), as well as the modified interaction network, we proposed a projection-based neighborhood non-negative matrix decomposition algorithm to predict the potential lncRNA-protein interactions. On two benchmark datasets, PMKDN showed better performance than other state-of-the-art methods for the prediction of new lncRNA-protein interactions, new lncRNAs, and new proteins. Case study further indicates that PMKDN can be used as an effective tool for lncRNA-protein interaction prediction.

## Introduction

RNA represents the direct output of genomic encoded genetic information, and a large part of the regulatory capacity of cells focuses on the synthesis, processing, transportation, modification, and translation of RNA. With the continuous improvement of RNA analysis, cell type isolation, and culture technology, people’s understanding of many biological functions of RNA is also getting higher and higher (DjebaliDavis and Merkel et al., 2012). Studies have shown that up to 85% of human genes are transcribed, but the proportion of RNA transcriptional codons encoding proteins is extremely low, suggesting that most RNA transcripts are non-coding (Fang and Fullwood, 2016). A large part of human genes plays their functions through non-coding RNA (ncRNA) (Mattick, 2005). Transcriptional ncRNA has similar chromosome modification functions to protein-coding genes. In multiple sites of human genome, the deletion of ncRNA will lead to the decline of the specificity of adjacent protein-coding genes (Ulf Andersson ørom et al., 2010). Long non-coding RNA (lncRNA) is an important type of ncRNA, which has more than 200 nucleotide transcripts and no obvious protein coding function (Volders et al., 2013). With the development of biological information, people are becoming more and more aware of the important role of lncRNA in various biological processes; lncRNA is involved in the regulation of gene expression and function of multiple networks, affects the formation of the kernel structure domain and whole chromosome state of transcription, and participates in the interaction of two different chromosomal regions through direct mechanisms regulating the chromosome structure (Batista and Chang, 2013). In addition, a growing number of studies have shown that mutations and disorders of lncRNA are associated with different human diseases. The primary structure, secondary structure, expression level of lncRNA, and changes in its homologous binding protein can lead to a variety of diseases ranging from neuropathy to cancer (Wapinski and Chang, 2011). Currently, more and more lncRNA have been discovered, but their functions and mechanisms are still poorly understood. In general, almost all lncRNA functions are expressed through the interaction with the corresponding RNA-binding proteins, and their functions and mechanisms depend on their interaction with various protein complexes in cells (Khalil and Rinn, 2011). Therefore, it is important to determine the potential interactions between lncRNAs and proteins to study the functions of lncRNA. It is expensive and time-consuming to detect large-scale lncRNA-protein interactions by experimental means, so a large number of computational models are proposed based on existing experimental data (Suresh et al., 2015).

Based on the physicochemical properties of peptide chains and nucleotide chains, Bellucci et al. (2011) proposed catRAPID in 2011, which combined secondary structure, hydrogen bonding, and van der Waals to predict the interactions between lncRNAs and proteins. Subsequently, Lu et al. (2013) proposed the lncPro model, which used the secondary structure, hydrogen bonds, van der Waals, and other features to encode nucleotide and amino acid sequences into feature vector, and calculated the interaction scores between lncRNAs and proteins by Fisher’s linear discriminant method. Suresh et al. (2015) proposed the RPI-Pred to predict the interactions between lncRNAs and proteins, which combined the secondary structural feature of RNA sequences with the three-dimensional structural feature of proteins and used support vector machine (SVM) model for prediction. Xiao et al. (2017) proposed a PLPIHS model, which constructed a heterogeneous model by using lncRNA-lncRNA similarity network, lncRNA-protein interaction network, and protein-protein interaction network, and then established a SVM classifier to predict lncRNA-protein interaction by HeteSim score. Subsequently, Deng et al. (2018) improved on PLPIHS and proposed a PLIPCOM model, which simultaneously obtained the low-dimensional features of lncRNA (protein) by restarted random walk and singular value decomposition on heterogeneous networks, and then used the gradient asymptotic tree algorithm to predict by combining the HeteSim score and low-dimensional features. Both algorithms achieved high AUC values, but they used the known lncRNA-protein interaction information to construct heterogeneous network, which also led to the reuse of the known interactions. Recently, Hu et al. (2018) proposed an ensemble strategy to predict potential lncRNA-protein interactions (HLPI-Ensemble), which used the strategy of random pairing to generate negative samples of lncRNA-protein interactions, and integrated support vector machine (SVM), random forest (RF), and extreme gradient enhancement (XGB) three mainstream machine learning algorithms to predict interaction scores. This ensemble learning strategy can not only improve the prediction performance of the model, but can also prevent the over-fitting of the model to some extent. Pan and Shen. (2017) used hybrid convolutional neural network and deep belief network to predict RNA-protein binding sites on RNAs, which used multimodal deep learning to fuse shared features of different sources of data, and found the explainable binding motifs. The above supervised learning method has achieved certain effects in predicting lncRNA-protein interactions, but there are still some problems. First, the key to supervised learning is to construct as balanced as possible positive and negative samples, but at present, most databases only provide lncRNA-protein interaction information, while the construction of negative samples is still a problem. Second, lncRNA-protein interaction prediction problem is a serious unbalanced classification problem, and the known interaction accounts for less than 1% of the total lncRNA-protein pairs, while many supervisory models often choose the same number of positive and negative samples as training set and test set, which artificially reduces the prediction range of the model to some extent. Finally, both lncRNA and protein exist in a whole biological network, and the rational use of lncRNA (protein) network topology can greatly improve the predictive performance of the model.

Recently, many network-based models have been proposed for predicting lncRNA-protein interactions. Li et al. (2015) proposed a heterogeneous network model to predict lncRNA-protein interactions, which constructed a lncRNA similarity network using lncRNA expression profiles and protein similarity network using weighted protein-protein interactions (PPIs), then combined with known lncRNA-protein interaction network uses the restart random walk model to make predictions. Ge et al. (2016) proposed a binary network inference algorithm (LPBNI) using only the known lncRNA-protein interactions to infer potential lncRNA-associated proteins. Zheng et al. (2017) predicted potential lncRNA-protein interactions by fusing multiple network information. Specifically, based on protein sequence, protein domain, protein GO term and STRING dataset, the method constructed four protein similarity networks, respectively, and integrated with similarity network fusion algorithm (SNF), and then used random walk algorithm to calculate the score. Recently, Zhang et al. (2018a) proposed a linear neighborhood propagation algorithm (LPLNP) to predict the potential lncRNA-protein interactions. Specifically, based on various feature extracted, LPLNP calculated the linear neighborhood similarity of the corresponding lncRNA (protein), and used the label propagation algorithm to calculate the interaction scores, and finally the linear combination of all prediction scores as the final result. Subsequently, Zhang et al. (2018b) proposed a sequence-based feature projection ensemble learning algorithm (SFPEL-LPI). Specifically, based on lncRNA sequences, protein sequences, and known lncRNA-protein interactions, SFPEL-LPI extracted a variety of lncRNA (protein) features and similarity information, and uses feature projection ensemble learning framework to predict lncRNA-protein interaction scores. Compared to LPLNP, SFPEL-LPI has fewer parameters and higher precision and can predict new lncRNAs and new proteins. Most network-based models build similarity networks by mining lncRNA (protein) related information and use their network topological structure and known lncRNA-protein interaction information for prediction and have the advantage of not requiring negative sample construction. In addition, this type of method is also global; based on the prediction results, we can get the prediction ranking of all unknown interaction pairs, which is more convenient for us to study the higher-ranking unknown interaction. However, in addition to SFPEL-LPI, other network-based methods only focus on the construction of similarity networks and ignore important feature information. Although SFPEL-LPI makes use of both feature information and similarity information, it separates the lncRNA network and protein network for prediction, which also limits the improvement of model performance.

Based on this, this study proposes a projection-based neighborhood non-negative matrix factorization (PMDKN) to predict potential lncRNA-protein interactions in heterogeneous omics data, which is also applicable to the prediction of new lncRNAs and new proteins. First, based on the lncRNA sequences, lncRNA expression profile, and protein sequences, we extracted a variety of features of lncRNA and protein. Second, based on multiple features of lncRNA and protein, lncRNA sequences, gene ontology annotation of the protein and the modified lncRNA-protein interaction network, we calculated multiple similarities of lncRNA and protein and fused to obtain more accurate lncRNA (protein) similarity network. Finally, PMDKN uses these features and fused similarity network to predict lncRNA-protein interaction scores. The results indicate that PMDKN exhibits higher predictive performance than other state-of-the-art methods for the prediction of lncRNA-protein interactions, new lncRNAs, and new proteins. Case study further demonstrates that PMDKN can be an effective tool for lncRNA-protein interaction.

## Materials and Methods

### Dataset

The noncoding RNAs and protein related biomacromolecules interaction database (Npinter) (Wu et al., 2006) provides a large number of experimentally verified interactions between non-coding RNA and other biomolecules. So far, Npinter has been updated to version 3.0, which includes more lnRNA-protein interactions than the previous version (Hao et al., 2016). In order to evaluate the predictive performance of the algorithm, we performed cross-experiment using the interactive data provided in Npinter v2.0 (Yuan et al., 2013) as the benchmark dataset and used Npinter v3.0 to test the final prediction ability of the model. Li et al. (2015) extracted interactions from Npinter v2.0 by limiting the organization to ‘Homo sapiens’ and ncRNA to ‘NONCODE’ and processed 4,870 interactions between 1,113 lncRNAs and 96 proteins. On this basis, Zhang et al. (2018a) deleted lncRNAs and proteins with no sequence information and only one interaction, resulting in 4,158 interactions between 990 lncRNAs and 27 proteins. Meanwhile, various features and similarity information were extracted based on the sequence data of lncRNAs and proteins. In order to facilitate the experimental comparison, we used the dataset provided by Zhang et al. (2018a) as the benchmark DATASET 1 for verification.

In benchmark DATASET 1, all lncRNAs (proteins) interact with at least two proteins (lncRNAs), and the number of lncRNA-protein interactions is relatively dense. To investigate the predictive ability of the algorithm for sparse interactions, lncRNAs without sequence information were deleted from the data provided by Li et al., and a total of 4,679 interactions between 1,068 lncRNAs and 90 proteins were finally obtained. Meanwhile, sequence information of lncRNA and expression profile information of lncRNA in 24 human tissues and cells were extracted from the integrated knowledge database of non-coding RNAs database (NONCODE) (Liu, 2004; Xie et al., 2013; Fang et al., 2018), and sequence information of protein and gene ontology annotation of protein were extracted from the protein-protein interaction networks dataset (STRING 9.1) (Franceschini et al., 2012). Based on the relevant information of lncRNA and proteins, multiple features and similarities of lncRNA (proteins) were calculated to construct benchmark DATASET 2.

### Features for lncRNAs and Proteins

Let $ℒ=\left\{l_{1},l_{2},\cdots ,l_{N_{l}}\right\}$ and $=\left\{p_{1},p_{2},\cdots ,p_{N_{p}}\right\}$ represent the set of *N_l_* lncRNAs and *N_p_* proteins obtained, respectively. In this section, we introduce the three features of lncRNA, the two features of the protein, and the similarity of lncRNA and the similarity of protein.

#### Features of lncRNA

We extracted three features of lncRNA, namely expression profile feature and two sequence-based features: pseudo-k-tuple nucleotide composition (PseKNC) (Chen et al., 2014) and parallel related pseudo dinucleotide composition (PCPseDNC) (Guo et al., 2014). For lncRNA, k-mer (nucleotide sequence of length k) is generally used to describe the short-term ordered information of the sequences, while the overall or long-term information of the sequences is described by the physicochemical properties of nucleotides. PseKNC and PCPseDNC describe the lncRNA by integrating the short-term and long-term features of the sequences (Chen et al., 2014). We calculated the PseKNC and PCPseDNC of lncRNA using python “repDNA” package (Liu et al., 2015).

#### Features of Protein

The hydrophilicity and hydrophobicity of proteins play an important role in protein folding, environmental and molecular interactions, and catalytic effects. Combining the frequency of regularization of 20 amino acids in the protein sequence and the distribution pattern of hydrophilicity and hydrophobicity along the protein chain, we calculated the characteristics of the two proteins, which are the amphiphilic pseudo amino acid composition (APseAAC) (Chou, 2001; Chou, 2005) and the combined triad descriptor (CTriad). Among them, Ctriad was proposed by Shen et al. (2007) to predict protein-protein interactions. First, in order to reduce the size of the feature space, 20 amino acids were grouped into 7 classes according to the dipole and volume of the side chains. Second, using the classes of amino acids to distinguish any conjoint triad (combination of any three consecutive amino acids) and counting the frequency. *f*(*v_i_*)*i*=1,2,.···,7^3^ of the occurrence of the conjoint triad in the amino acid sequence, where *v_i_* represents the *i*-th conjoint triad. Finally, normalizing *f*(*v_i_*), we could get the conjoint triplet descriptor feature CTriad(P)=[*q*_1_,*q*_2_,···,*q*_343_] of protein P as follows:

$$q_{i}=\frac{f\left(v_{i}\right)-\min{\left\{f\left(v_{i}\right)\right\}}_{i=1}^{343}}{\max{\left\{f\left(v_{i}\right)\right\}}_{i=1}^{343}-\min{\left\{f\left(v_{i}\right)\right\}}_{i=1}^{343}}$$

Where, $\min{\left\{f\left(v_{i}\right)\right\}}_{i=1}^{343}$ and $\max{\left\{f\left(v_{i}\right)\right\}}_{i=1}^{343}$ represent the minimum and maximum frequencies of all conjoint triads, respectively. It should be noted that in order to prevent the over-fitting problem caused by the lncRNA (protein) feature due to the high dimension, we use the PCA for dimensionality reduction on the high-dimensional features.

### Similarities for LncRNAs and Proteins

In this section, we introduce the lncRNA-lncRNA similarity and the protein-protein similarity.

#### lncRNA-lncRNA Sequence Similarity

Kirk et al. (2018) found that lncRNAs with related functions, although lacking linear homology, often have a similar k-tuple spectrum, which is related to lncRNA binding protein and its subcellular localization. Song et al. (2014) introduced a variety of alignment-free genome and metagenome comparison methods based on word frequency and proved that $d_{2}^{*}$ has a stronger statistical ability to measure sequence correlation. Therefore, $d_{2}^{*}$ was used in this study to calculate the sequence similarity between lncRNAs. For any two lncRNA sequences *L*_1_ and *L*_2_ with *m* and n nucleotides, respectively, the dissimilarity $d_{2}^{*}\left(L_{1},L_{2}\right)$ is as follows:

$$d_{2}^{*}\left(L_{1},L_{2}\right)=\frac{1}{2}\left(1-\frac{D_{2}^{*}\left(L_{1},L_{2}\right)}{\sqrt{\sum_{w\in A^{k}}{\overset{˘}{X}}_{w}^{2}/\left(\bar{m}p_{w}^{X}\right)}\sqrt{\sum_{w\in A^{k}}{\overset{˘}{Y}}_{w}^{2}/\left(\bar{n}p_{w}^{Y}\right)}}\right)$$

Where $D_{2}^{*}\left(L_{1},L_{2}\right)$ represents the $D_{2}^{*}$ statistic of *L*_1_ and *L*_2_, and $p_{w}^{X}$ and $p_{w}^{Y}$ respectively represent the probability of *k*-tuple w occurring in *L*_1_ and *L*_2_ of lncRNA under the background model. $\bar{m}=m-k$ , $\bar{n}=n-k$ , ${\overset{˘}{X}}_{w}=X_{w}-\bar{m}p_{w}^{X}$ , ${\overset{˘}{Y}}_{w}=Y_{w}-\bar{n}p_{w}^{Y}$ , where *X_w_* and *Y_w_* represent the frequencies at which the k-tuples in the sequences *L*_1_ and *L*_2_ occur, respectively. Further, the similarity of *L_1_* and *L_2_* is $\left(1-d_{2}^{*}(L_{1},L_{2})\right)$ . We used the program provided by Ahlgren et al. (2016) to calculate the $d_{2}^{*}$ similarity of lncRNA.

#### Protein-Protein Semantic Similarity

The semantic comparison of gene ontology annotations provides a quantitative method for calculating the semantic similarity of gene products (Yu et al., 2010). There are currently two classic methods for computing the semantic similarity of GO annotation items: information-based methods (Jiang and Conrath, 1997; Lin, 1998; Resnik, 1999) and graph-based (Wang et al., 2007) methods, respectively. In this study, the graph-based method was first used to calculate the semantic similarity of GO items, and then the semantic similarity of proteins was calculated according to the association between protein and GO items. Specifically, any GO item A could be expressed as DAG(A)=(A,*T_A_*,*E*_A_), where *T_A_* represents the set containing item A and all its ancestor items in the GO diagram, and *E_A_* represents the set connecting all edges of GO item in DAG(A). Then, for any two GO annotation items A and B, their semantic similarity could be defined as:

$$\text{S}\left(\text{A},\text{B}\right)=\frac{\sum_{t\in T_{A}\cap^{}T_{B}}S_{A}\left(t\right)+S_{B}\left(t\right)}{SV\left(A\right)+SV\left(B\right)}$$

Where, *S_A_*(*t*) and *S_B_*(*t*) represent the S-value of GO item t related to item A and item B respectively, and $SV\left(A\right)=\underset{t\in T_{A}}{\sum }S_{A}\left(t\right)$ represents the semantic value of GO item A. At this point, according to the correlation between protein and GO term, we can get the semantic similarity of protein. We use R package “protr” to obtain semantic similarity of proteins; more details are shown in (Xiao et al., 2015).

#### Kernel Neighborhood Similarity

In Section “Features for lncRNAs and Proteins”, we obtained three features of lncRNA and two features of protein, and the known lncRNA-protein interaction network also contains important lncRNA (protein) feature information. Based on these feature vectors, there are many methods for calculating similarities, such as Gaussian, linear neighborhood similarity (Zhang et al., 2018a) (LNS), and so on. Here, we adopt kernel neighborhood similarity (KSNS) (Ma et al., 2018a; Ma et al., 2018b), which not only considers the neighbor and non-neighbor similarity of samples hierarchically, but also explores nonlinear relations, which was well applied to a variety of biological problems. It should be noted that the currently known lncRNA-protein interaction matrix is incomplete. Therefore, in order to reduce the error caused by information loss, we first use the Weighted K nearest neighbor profiles (WKNNP) (Xiao et al., 2018) to complete the known interaction matrix, and then calculated the KSNS of lncRNA(protein) interaction profile.

**Figure 1:**
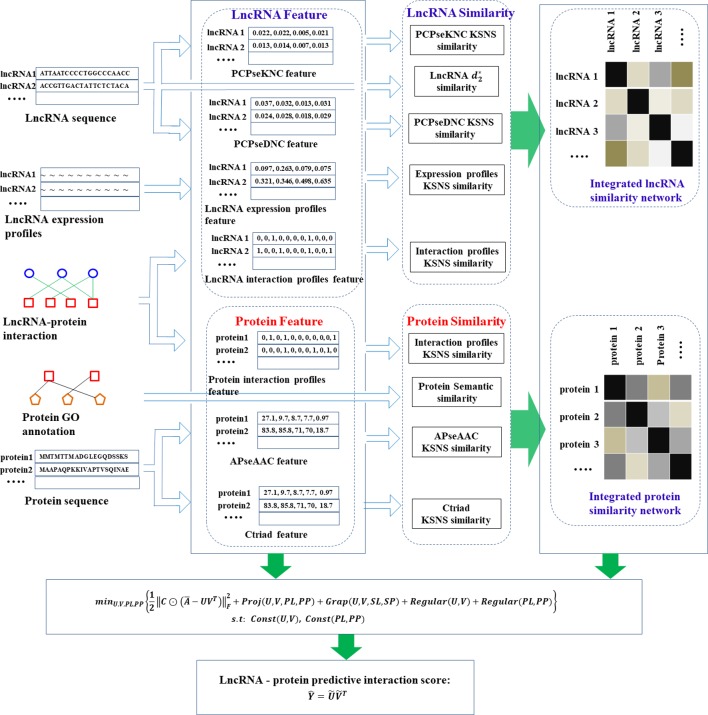
Flow chart of lncRNA-protein interaction prediction by PMDKN algorithm. As shown in the figure, we first calculated three features of lncRNAs and two features of proteins, and then calculated five similarities of lncRNAs and four similarities of proteins according to lncRNA sequence, protein GO annotation and their features.

Based on the above steps, we obtained a total of 5 similarities of lncRNAs and 4 similarities of proteins, which reflected the similarity relationship of lncRNAs (proteins) from different perspectives. Due to the limitations of data and the selection of computational methods, these similarity networks may contain noise. Hence, we adopted a clusDCA proposed by Wang et al. (2015) for similarity network fusion, which can not only eliminate network noise and effectively capture network topology, but also have high computational efficiency in large-scale networks. The general procedure for predicting lncRNA-protein interaction using PMDKN is shown in **Figure 1**.

### Prediction of lncRNA-Protein Interaction

Based on various features of lncRNA (protein) and the integrated lncRNA (protein) similarity network, we proposed projection-based neighborhood non-negative matrix factorization (PMDKN) to predict potential lncRNA-protein interactions. ${\left\{FL_{i}\right\}}_{i=1}^{N_{1}}$ represents the *N*_1_ feature matrices of lncRNA, ${\left\{FP_{i}\right\}}_{i=1}^{N_{2}}$ represents the *N*_2_ feature matrices of protein, similarity matrix of lncRNA and protein are SL and SP respectively, A represents known lncRNA-protein interaction matrix, and $\bar{A}$ represents lncRNA-protein interaction matrix completed by WKNNP.

First, we mapped lncRNA and protein to the common non-negative space *R^d^*, that is, any lncRNA *l_i_* and protein *p_j_* can be represented by non-negative latent vectors $u_{i}\in R^{d\times 1}$ and $v_{j}\in R^{d\times 1}$ . For simplicity, we further denote the latent vectors of all lncRNAs and all proteins by $U={\left(u_{1},\cdots ,u_{N_{l}}\right)}^{T}\in R^{N_{l}\times d}$ and $\text{V}=\left(v_{1},\cdots ,v_{N_{h}}\right)\in R^{d\times N_{p}}$ , then, the product of the U and V can be used to approximate the modified interaction matrix *Ā*. Since the observed interactions have been verified by experiments and have higher reliability than the unknown interactions, the observed lncRNA-protein interactions are assigned a higher level of importance and can be obtained as follows:

$$min_{U,V}\left\{\frac{1}{2}||C⊙\left(\bar{A}-UV^{T}\right)|{|}_{F}^{2}+\frac{\gamma }{2}\left(||U|{|}_{F}^{2}+||V|{|}_{F}^{2}\right)\right\}$$

(1) s.t.   U≥0, V≥0

where *C* is the importance level distribution matrix, that is, if there is interaction between the lncRNA *l_i_* and the protein *p_j_*, *C_i,j_*= *δ*, otherwise, *C_i,j_* = 1, where δ > 1 is an important level parameter. **||**·**||***_F_* denotes the *F*-norm and *γ* denotes the regularization parameter of latent vectors.

In addition, in order to integrate different types of lncRNA features, we project all lncRNA features onto the non-negative space *R^d^*, and required the difference between it and U to be as small as possible, so as to obtain:

$$\min_{\;PL_{i}}\left\{\sum_{i=1}^{N_{1}}\alpha_{i}^{\eta }||FL_{i}PL_{i}^{T}-U|{|}_{F}^{2}+\frac{\mu }{2}\sum_{i=1}^{N_{1}}\sum_{k=1}^{r}||PL_{i}(k,:)|{|}_{1}^{2}\right\}$$

(2) $$\text{s.t.}\;PL_{i}\geq 0$$

where $FL_{i}\in R^{N_{l}\times d_{l_{i}}}$ represents the *i*-th feature matrix of lncRNA, *d_li_* represents the dimension of the feature, and $PL_{i}\in R^{r\times d_{l_{i}}}$ represents the corresponding projection matrix. In order to facilitate calculation and convenient interpretation, *PL_i_* is required to be non-negative. The Weight vector $\alpha =\left(\alpha_{1},\alpha_{2},\cdots ,\alpha_{N_{1}}\right)$ controls the effect of different feature projections on U. The projection index parameter *η* > 1 is the index of *α*, indicating that all features contribute to the generation of U. µ is the regularization parameter of projection matrix, and *P*(*k*,:) is the *k*-th row of the matrix *P*. ||*PL_i_*(*k*,:)|| represents the $\ell_{1}$ -norm of the vector *PL_i_*(*k*,:) (ie, $||P\left(k,:\right)|{|}_{1}=\underset{j}{\sum }\left|P\left(k,j\right)\right|$ ), ensuring that the projection vector *PL_i_*(*k*,:) is as sparse as possible, and $\overset{r}{\underset{k=1}{\sum }}||PL_{i}\left(k,:\right)|{|}_{1}^{2}$ is equivalent to the square of the $\ell_{1,2}$ -norm of the matrix *PL_i_*. Therefore, equation (2) can be expressed as follows:

$$\min_{\;PL_{i}}\left\{\sum_{i=1}^{N_{1}}\alpha_{i}^{\eta }||FL_{i}PL_{i}^{T}-U|{|}_{F}^{2}+\frac{\mu }{2}\sum_{i=1}^{N_{1}}||PL_{i}|{|}_{1,2}^{2}\right\}$$

(3) $$s.t.\;PL_{i}\geq 0$$

Similarly, for proteins, we have:

$$\min_{\;PP_{j}}\left\{\sum_{j=1}^{N_{2}}\beta_{j}^{\eta }||FP_{j}PP_{j}^{T}-V|{|}_{F}^{2}+\frac{\mu }{2}\sum_{j=1}^{N_{2}}||PP_{j}|{|}_{1,2}^{2}\right\}$$

(4) $$\text{s.t.}\;PP_{j}\geq 0$$

where $FP_{j}\in R^{N_{p}\times d_{p_{j}}}$ represents the *j*-th feature matrix of the protein, and non-negative matrix $PP_{j}\in R^{r\times d_{p_{i}}}$ represents the corresponding projection matrix. The weight vector $\beta =\left(\beta_{1},\beta_{2},\cdots ,\beta_{N_{2}}\right)$ controls the effect of feature projection on V.

It is generally believed that lncRNAs with higher similarity are more likely to interact with the same protein, but due to the incomplete data set, the similarity network of lncRNAs (proteins) obtained may contain noise. In order to eliminate the influence of non-neighborhood noise and improve the prediction accuracy, we only consider strong neighborhood similarity relationship of the samples. Therefore, lncRNA neighborhood similarity matrix ($\bar{SL}$ ) was constructed as follows:

(5) $${\bar{SL}}_{i,j}=\{\begin{array}{c} SL_{i,j}\;\;\;\;\;\;\;\;\;\;\;if\;l_{j}\in N\left(l_{i}\right)\;or\;l_{i}\in N\left(l_{j}\right)\\ 0\;\;\;\;\;\;\;\;\;\;\;\;\;otherwise\;\;\;\;\;\;\;\;\;\;\;\;\;\;\;\;\;\;\;\;\;\;\; \end{array}$$

Among them, ${\bar{SL}}_{i,j}$ represents the local similarity of lncRNA *l_i_* and *l_j_*, and *N*(*l_i_*) represents the K neighbor sets closest to lncRNA *l_i_*. In order to adaptively select the number of neighbors according to the sample size, we make $K=0.3\times N_{l}$ ,  ⌈⋅⌉ indicates rounding up. It is known from equation (5) that $\;\bar{\text{SL}}$ is a symmetric matrix. According to lncRNAs with higher similarity, their features are as close as possible; we have:

(6) $$\frac{\lambda }{2}\underset{i}{\sum }\underset{j}{\sum }{\bar{SL}}_{i,j}||u_{i}-u_{j}|{|}_{F}^{2}=\lambda tr\left(U^{T}\left(D_{l}-\bar{SL}\right)U\right)=\lambda tr\left(U^{T}LP_{l}U\right)$$

Where tr(⋅) represents the trace of the matrix, λ is the neighborhood Laplacian regularization parameter, and $LP_{l}=DL-\bar{SL}$ is the Laplacian matrix of the lncRNA. The diagonal matrix $D_{l}=diag\left(dL_{1},dL_{2},\cdots ,dL_{N_{l}}\right)$ , whose diagonal elements are $dL_{i}=\underset{k}{\sum }{\bar{SL}}_{i,k}$ , respectively. Similarly, we can calculate the neighborhood similarity matrix $\bar{SP}$ of the protein as follows:

(7) $${\bar{SP}}_{i,j}=\{\begin{array}{c} SP_{i,j}\;\;\;if\;p_{j}\in N\left(p_{i}\right)\;or\;p_{i}\in N\left(p_{j}\right)\\ 0\;\;\;\;\;\;\;\;\;\;\;\;\;otherwise \end{array}$$

Furthermore, the objective function can be obtained as follows:

(8) $$\frac{\lambda }{2}\underset{i}{\sum }\underset{j}{\sum }{\bar{SP}}_{i,j}{\left\|p_{i}-p_{j}\right\|}_{F}^{2}=\lambda tr\left(V^{T}\left(D_{p}-\bar{SP}\right)V\right)=\lambda tr\left(V^{T}LP_{p}V\right)$$

where $LP_{p}=D_{p}-\bar{SP}$ is the Laplacian matrix of the protein. The diagonal matrix $D_{p}=diag\left(dP_{1},dP_{2},\cdots ,dP_{N_{p}}\right)$ , whose diagonal elements are $dP_{i}=\underset{k}{\sum }{\bar{SP}}_{i,k}$ , respectively. Combined with the above formulas, the objective function of PMKDN algorithm can be obtained as follows:

$$\min_{U,V,GU_{i},GV_{j},\alpha_{i,}\beta_{j}}\{\frac{1}{2}||C⊙(\bar{A}-UV^{T})|{|}_{F}^{2}=\frac{1}{2}\sum_{i=1}^{N_{1}}\alpha_{i}^{\eta }||FL_{i}PL_{i}^{T}-U|{|}_{F}^{2}+\frac{1}{2}\sum_{j=1}^{N_{2}}\beta_{j}^{\eta }||FP_{j}PP_{j}^{T}-V|{|}_{F}^{2}+\frac{\lambda }{2}\left(tr(U^{T}LP_{l}U)+tr(V^{T}LP_{p}\text{V})\right)+\frac{\mu }{2}\left(\sum_{i=1}^{N_{1}}||PL_{i}|{|}_{1,2}^{2}+\sum_{j=1}^{N_{2}}||PP_{j}|{|}_{1,2}^{2}\right)+\frac{\gamma }{2}(||U|{|}_{F}^{2}+||V|{|}_{F}^{2})\}$$

(9) $$\begin{array}{c} st.\;\;\;U\geq 0,\;V\geq 0,\;\;PL_{i}\geq 0,\;PP_{j}\geq 0,\overset{N_{1}}{\underset{i=1}{\sum }}\alpha_{i}=1,\\ \overset{N_{2}}{\underset{j=1}{\sum }}\beta_{j}=1,\;\alpha_{i}\geq 0,\;\beta_{j}\geq 0 \end{array}$$

We use the two-step method to solve (9). First, by fixing *α_i_*, *β_j_*, and using the Lagrangin multiplier and the KKT condition, we can get the iterative formula of U, V, *PL_i_* and *PP_j_* as follows:

(10) $$\text{U}\;=U⊙\frac{\left(C⊙\bar{A}\right)V+\sum_{i=1}^{N_{1}}\alpha_{i}^{\eta }FL_{i}PL_{i}^{T}+\lambda_{1}\bar{SL}U}{\left(C⊙UV^{T}\right)V+\sum_{i=1}^{N_{1}}\alpha_{i}^{\eta }U+\lambda_{1}D_{l}\;U+\gamma_{1}U}$$

(11) $$\text{V}=V⊙\frac{\left(C⊙{\bar{A}}^{T}\right)U+\sum_{i=1}^{N_{1}}\beta_{j}^{\eta }FP_{j}PP_{j}^{T}+\lambda_{2}\bar{SP}V}{\left(C⊙VU^{T}\right)U+\sum_{j=1}^{N_{2}}\beta_{j}^{\eta }V+\lambda_{2}D_{p}V+\gamma_{2}V}$$

(12) $$PL_{i}=PL_{i}⊙\frac{\alpha_{i}^{\eta }U^{T}FL_{i}}{\alpha_{i}^{\eta }PL_{i}FL_{i}^{T}FL_{i}+\mu_{1}PL_{i}ee^{T}}$$

(13) $$PP_{j}=PP_{j}⊙\frac{\beta_{j}^{\eta }V^{T}FP_{j}}{\beta_{j}^{\eta }PP_{j}FP_{j}^{T}FP_{j}+\mu_{2}PP_{j}ee^{T}}$$

Then, fix U, V, *PL_i_* and *PP_j_*, and let $a_{i}=||FL_{i}PL_{i}^{T}-U|{|}_{F}^{2}\geq 0$ , $b_{j}=||FP_{j}PP_{j}^{T}-V|{|}_{F}^{2}\geq 0$ , *C*_1_ represents the terms unrelated to *α_i_* and *β_j_* (3.8). We can get the objective function about *α_i_* and *β_j_* as follows:

$$\min_{\alpha ,\beta }\left\{\frac{1}{2}\overset{N_{u}}{\underset{i=1}{\sum }}a_{i}\alpha_{i}^{\eta }+\frac{1}{2}\overset{N_{v}}{\underset{j=1}{\sum }}b_{j}\beta_{}^{\eta }+C_{1}\right\}$$

$$\overset{N_{u}}{\underset{i=1}{\sum }}\alpha_{i}=1,\overset{N_{v}}{\underset{j=1}{\sum }}\beta_{j}=1,\alpha_{i}\geq 0,\;\beta_{j}\geq 0$$

Using the Lagrangian multiplier, the iterative formula for *α_i_* and *β_j_* can be obtained as follows:

(14) $$\alpha_{i}=\frac{{\left(\frac{1}{a_{i}}\right)}^{\frac{1}{\eta -1}}}{\sum_{i=1}^{N_{1}}{\left(\frac{1}{a_{i}}\right)}^{\frac{1}{\eta -1}}}=\frac{{\left(\frac{1}{||FL_{i}PL_{i}^{T}-U|{|}_{F}^{2}}\right)}^{\frac{1}{\eta -1}}}{\sum_{i=1}^{N_{1}}{\left(\frac{1}{||FL_{i}PL_{i}^{T}-U|{|}_{F}^{2}}\right)}^{\frac{1}{\eta -1}}}$$

(15) $$\beta_{j}=\frac{{\left(\frac{1}{b_{j}}\right)}^{\frac{1}{\eta -1}}}{\sum_{j=1}^{N_{2}}{\left(\frac{1}{b_{j}}\right)}^{\frac{1}{\eta -1}}}=\frac{{\left(\frac{1}{FP_{j}PP_{j}^{T}-V_{F}^{2}}\right)}^{\frac{1}{\eta -1}}}{\sum_{j=1}^{N_{2}}{\left(\frac{1}{FP_{j}PP_{j}^{T}-V_{F}^{2}}\right)}^{\frac{1}{\eta -1}}}$$

According to (14) and (15), *α_i_* and *β_j_* always satisfy non-negative constraints. In formula (9), U and V are obtained based on the decomposition of the known lncRNA-protein interaction matrix. In order to reduce the prediction error of the new lncRNA (lncRNA without any protein interaction information) and the new protein, we utilized the method proposed by Liu et al. (2016), that is, the lncRNA(protein) was modified by using the neighborhoodlatent vectors. Let *ũ_i_* the modified latent vector of lncRNA *l_i_*, which can be calculated as follows:

(16) $${\tilde{u}}_{i}=\{\begin{array}{c} \;\;\;\;\;\;\;\;\;\;\;\;\;\;\;\;\;\;u_{i}\;\;\;\;\;\;\;\;\;\;\;\;\;\;\;\;\;\;\;\;\;\;\;\;\;\;\;\;\;\;\;\;if\;\;\;\;\overset{N_{p}}{\underset{j=1}{\sum }}A_{i,j}>0\\ \frac{1}{QL_{i}}\sum_{s\in N^{+}\left(l_{i}\right)}SL\left(i,s\right)u_{s}\;\;\;\;\;\;\;\;\;\;\;otherwise\;\;\;\;\;\;\;\;\;\;\;\;\;\;\; \end{array}$$

where, the first item $\sum_{j=1}^{N_{p}}A_{i,j}>0$ indicates that the latent vector of lnRNA with protein interaction remain unchanged. The second term refers to the modification of latent vector of lncRNAs without protein interaction, where *N*^+^(*l_i_*) refers to the set composed of K lncRNAs with the highest similarity to *l_i_* among lncRNA sets with protein interaction. In order to make the number of neighbors automatically adapt to the size of samples, we set $K=\text{max}\left(5,\left\lfloor 0.1\times N_{l}\right\rfloor \right)$ , where $Q_{i}=\sum_{s\in N^{+}\left(d_{i}\right)}SL\left(i,s\right)$ represents the normalized term. Similarly, we modified the latent vector of proteins as follows:

(17) $${\tilde{v}}_{j}=\{\begin{array}{c} \;\;\;\;\;\;\;\;\;\;\;\;v_{j}\;\;\;\;\;\;\;\;\;\;\;\;\;\;\;\;\;\;\;\;\;\;\;\;\;\;\;\;\;\;\;\;\;\;\;if\;\;\;\;\overset{N_{l}}{\underset{i=1}{\sum }}A_{i,j}>0\\ \frac{1}{QP_{j}}\underset{t\in N^{+}\left(l_{j}\right)}{\sum }SP\left(j,t\right)v_{t}\;\;\;\;\;\;\;otherwise\;\;\;\;\;\;\;\;\;\;\;\;\; \end{array}$$

By using the modified latent vector $\tilde{U}=\left[{\tilde{u}}_{1},{\tilde{u}}_{2},\cdots ,{\tilde{u}}_{N_{l}}\right]$ of lncRNA and the modified latent vector $\tilde{V}=\left[{\tilde{v}}_{1},{\tilde{v}}_{2},\cdots ,{\tilde{v}}_{N_{p}}\right]$ of protein, we can obtain the final lncRNA-protein interaction score $\bar{Y}=\tilde{U}{\tilde{V}}^{T}$.

### Algorithm

In the process of model derivation, we assume that the features of lncRNA and protein are non-negative, so the original features need to be normalized before algorithm calculation. Let $\hat{F}\in R^{N\times M}$ represent the original feature matrix of lncRNA (protein), where ${\hat{F}}_{i,j}$ represents the *j*-th dimension of the *i*-th sample, then the normalized feature matrix F is as follows:

(18) $$F_{i,j}=\frac{{\hat{F}}_{i,j}-\min\left({\hat{F}}_{.j}\right)}{\max({\hat{F}}_{.j})-\min\left({\hat{F}}_{.j}\right)}$$

Where, min (*F*.*_j_*) and max (*F*.*_j_*) represent the minimum and maximum of the *j*-th dimension, respectively. Algorithm 1 summarizes the general process of solving lncRNA-protein interaction prediction by KDMPN.

Algorithm 1 **KDMPN.**

Input: Known lncRNA-protein interaction matrix *A*; Modified lncRNA-protein interaction matrix Ā; Importance level parameter δ; LncRNA original feature matrix ${\left\{\hat{FL_{l}}\right\}}_{i=1}^{N_{1}}$; Protein initial feature matrix ${\left\{\hat{FP_{j}}\right\}}_{j=1}^{N_{2}}$; LncRNA similarity matrix *SL*; Protein similarity matrix *SP*; Potential subspace regularization parameter *r*; Projection index parameter *η*>1; Projection matrix regularization parameter µ; Neighborhood Laplacian regularization parameter λ; Potential subspace regularization parameter γ.

Output: LncRNA latent vector Ũ; Protein latent vector $\tilde{V}$; Predictive interaction matrix $\bar{Y}$; LncRNA feature projection matrix ${\left\{PL_{i}\right\}}_{i=1}^{N_{1}}$; Protein feature projection matrix ${\left\{PP_{j}\right\}}_{j=1}^{N_{2}}$; LncRNA projection parameter ${\left\{\alpha_{i}\right\}}_{i=1}^{N_{1}}$; Protein projection parameter ${\left\{\beta_{j}\right\}}_{j=1}^{N_{2}}$.

Initialize:

1 The importance level distribution matrix ${\left(C\right)}_{N_{l}\times N_{p}}$ is calculated from δ and *A*, andthe normalized lncRNA feature matrix ${\left\{FL_{i}\right\}}_{i=1}^{N_{1}}$ and the protein projection matrix ${\left\{FP_{j}\right\}}_{j=1}^{N_{2}}$ are obtained by using Equation (18) for ${\left\{\hat{FL_{i}}\right\}}_{i=1}^{N_{1}}$ and ${\left\{\hat{FP_{l}}\right\}}_{i=1}^{N_{2}}$. Based on *SL* and *SP*, the neighborhood similarity matrices $\bar{SL}$ and $\bar{SP}$ of lncRNA and protein were obtained using equations (5) and (7), respectively. Initialize $U,V||\;{\left\{PL_{i}\right\}}_{i=1}^{N_{1}}$ and ${\left\{PP_{j}\right\}}_{j=1}^{N_{2}}$ using the random number of the [0, 1] interval.

2 for $i\leftarrow 1,2,\cdots ,N_{1}$ do

Fix *PL_i_* and U, calculate *α_i_* according to formula (14).

end for

for $i\leftarrow 1,2,\cdots ,N_{2}$ do

Fix *PP_j_* and V, calculate *β_j_* according to formula (15).

end for

**repeat**

3 Fix ${\left\{\alpha_{i}\right\}}_{i=1}^{N_{1}}$ and ${\left\{PL_{i}\right\}}_{i=1}^{N_{1}}$, Update *U* according to formula (10).

4 Fix ${\left\{\beta_{j}\right\}}_{j=1}^{N_{2}}$ and ${\left\{PP_{j}\right\}}_{j=1}^{N_{2}}$, Update *V* according to formula (11).

5 for $i\leftarrow 1,2,\cdots ,N_{1}$ do

Fix ${\left\{\alpha_{i}\right\}}_{i=1}^{N_{1}}$ and *U*, Update *PL_i_* according to formula (12).

Fix *PL_i_* and *U*, Update *α_i_* according to formula (14).

end for

6 for $j\leftarrow 1,2,\cdots ,N_{2}$ do

Fix ${\left\{\beta_{j}\right\}}_{j=1}^{N_{2}}$ and *V*, Update *PP_j_* according to formula (13).

Fix *PP_j_* and *V*, Update *β_j_* according to formula (15).

end for

**until Converges**

7 Ũ was obtained by completing the subspace feature *U* of lncRNA according to formula (16).

8 $\tilde{V}$ was obtained by completing the subspace feature *V* of protein according to formula (17).

9 $\bar{Y}=\tilde{U}{\tilde{V}}^{T}$

## Results and Discussion

### Experimental Settings

According to previous studies, the performance of the interactive prediction method was evaluated by the 5-fold cross validation (CV), and the area under ROC curve (AUC), area under Precision-Recall curve (AUPR), and F1 value (F1) were used as evaluation indexes. Since the known lncRNA-protein interactions were much less than the unknown lncRNA-protein interactions, AUPR was usually used as the most important evaluation index to punish false positives (Zhang et al., 2018a; Zhang et al., 2018b).

In addition, in order to eliminate the influence of random partition on the results in the crossover experiment, we selected the method ofLiu et al. (2016), set 5 random seeds for CV, and took the mean value of the cross experiment results under all random seeds as the final prediction result. Specifically, the lncRNA-protein interaction matrix $A\in R^{N_{l}\times N_{p}}$ has *N_l_* rows for lncRNAs and *N_p_* columns for proteins. In order to investigate the prediction ability for lncRNA-protein interactions, new lncRNAs and new proteins, we performed CV under three different settings, as follows:

1. *CV_a_*: CV on known lncRNA-protein interaction pairs. Specifically, we randomly divided the known lncRNA-protein interactions into 5 equal parts. Take turns to select one and all the unknown interactions to form the test set and the remaining four and all the unknown interactions to form the training set (that is, change the 1 corresponding to the test set in A into 0 as the training set).
2. *CV_l_*: CV on lncRNAs. Specifically, all lncRNAs are randomly divided into five equal parts; one is selected as a test set in turn, and the remaining four are training sets (that is, all the rows corresponding to the test set in A were changed to zeros).
3. *CV_p_*: CV on proteins. Specifically, all proteins are randomly divided into five equal parts; one is selected as a test set in turn, and the remaining four are training sets (that is, all the columns corresponding to the test set in A were changed to zeros).

It should be noted that with regard to *CV_a_*, we selected all zeros in *A* as the test set. For example, for DATA2, the test set of each crossover experiment contains 4,870/5 = 947 known interactions and 97,658 unknown interactions (that is, the ratio of positive and negative examples is approximately 1:100). This selection method ensures that all the unknown interactions can be included in each crossover experiment, which expands the search range and is also in line with the actual situation.

### Parameter Setting

The PMDKN algorithm have six parameters, namely the projection index parameter *η*, the projection regularization parameter µ, the latent vector regularization parameter *γ*, the neighborhood Laplacian regularization parameter *λ*, the potential subspace dimension *d*, and the known interaction important level parameter δ. Among them, µ and *γ* control the influence of feature projection, *γ* controls subspace feature contribution, *λ* describes the effect of similarity network, and δ controls the importance level of observed interaction. In order to study the effect of parameters on the prediction results, we calculated all the parameter combinations. Specifically, *η* was selected from {2,3,4,5,6}, µ was selected from {10^-3^,10^-2^,10^-1^,10^0^,10^1^}, γ was selected from {10^-3^,10^-2^,10^-1^,10^0^,10^1^}, and *λ* was selected from {2^-2^,2^-1^,2^0^2^1^,2^2^,2^3^}; according to the previous research (Zheng et al., 2013, Liu et al., 2016, Xiao et al., 2018), for methods based on matrix decomposition, the potential subspace dimension *d* = 100, δ was selected from {1,2,⋯, 6}.

**Figure 2:**
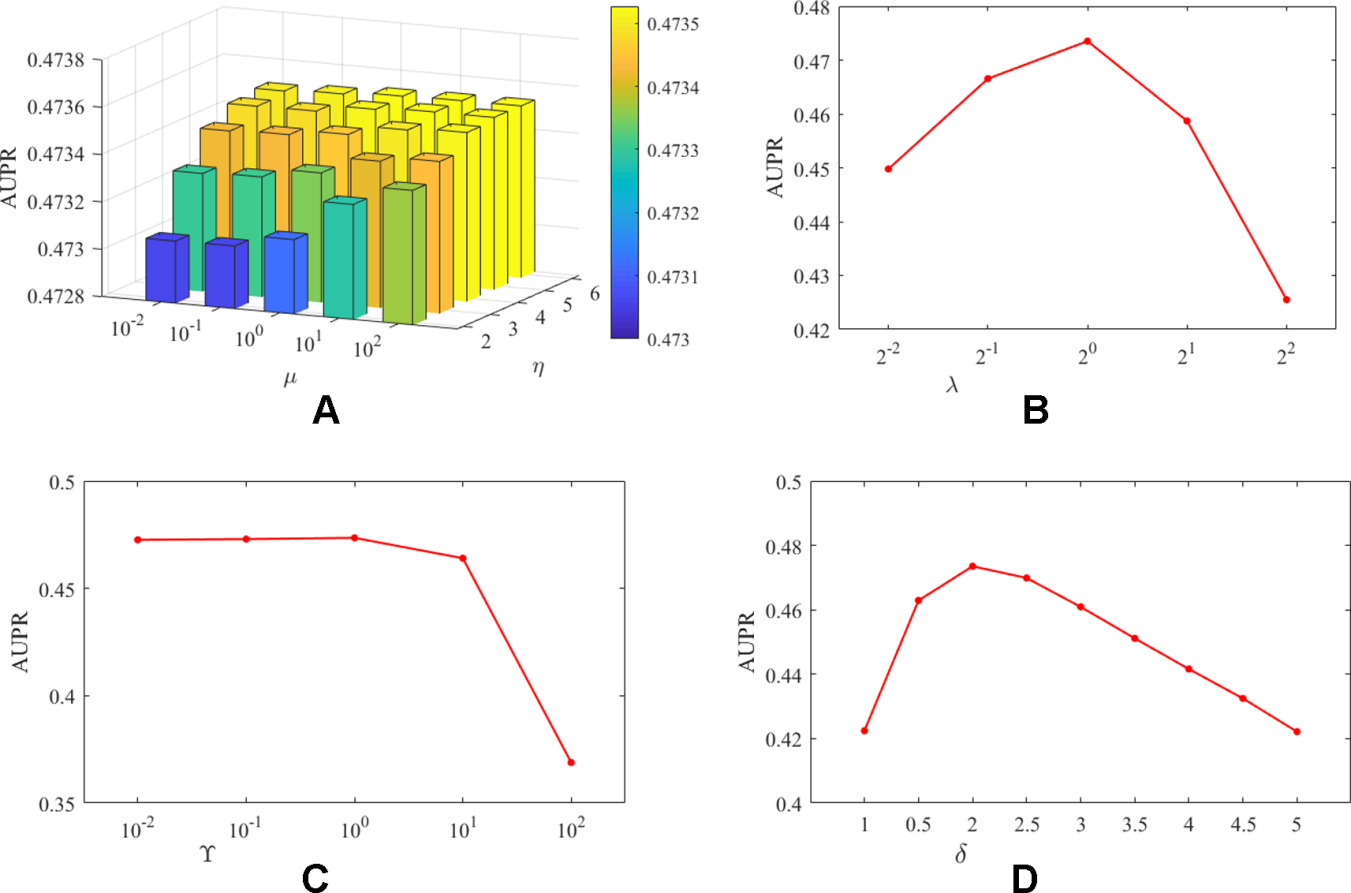
The influence of parameters on the AUPR value of PMDKN. Among them, **(A)** represents the influence of the projection parameters µ and η on the AUPR value. **(B)** shows the effect of the neighborhood Laplacian regularization parameter λ. **(C)** shows the effect of the feature regularization parameter *γ*. **(D)** indicates the effect of observing the important level parameter δ.

It should be noted that unlike DATASET 1, DATASET 2 contained more lncRNAs and proteins, and the initially constructed lncRNA (protein) similarity network did not utilize any known interaction information and therefore has higher predictive value. In addition, since *CV_a_*, *CV_l_*, and *CV_p_* are considered the predictive power of the algorithm for new interactions, new lncRNAs, and new proteins, respectively, we believe that the three experimental setups are equally important for algorithm evaluation. Therefore, based on DATASET 2, for the combination of different parameters, the average evaluation index of the algorithm under the three experimental settings is the final evaluation standard. We take AUPR as the evaluation index, and the influence of the analysis parameters on the prediction results was shown in **Figure 2**.

As shown in **Figure 2**, the optimal parameters obtained by the PMDKN algorithm are η = 5, µ = 100, λ = 1, γ = 1, δ = 2, and the average optimal AUPR value under the three experimental settings is 0.4735. Specifically, we first analyze the influence of the projection parameters *η* and µ. Fixed λ = 1, γ = 1, δ = 2, and calculate the AUPR value of the model under all possible combinations of *η* and µ. As shown in (A) of **Figure 2**, as *η* becomes larger, the AUPR value of the model increases, but the overall AUPR value of the model fluctuates a little. Then, we fixed *η* = 5, µ = 100, γ = 1, δ = 2, and analyzed the influence of the change of λ on the AUPR value. As shown in (B) of **Figure 2**, when λ increases, the AUPR value of the model first becomes larger and then decreases, and when λ = 1, the AUPR value is the largest. Similarly, as shown in (C) in **Figure 2**, when γ < 1, the change of AUPR was relatively flat; when γ > 1, the AUPR value decreased sharply with the increase of gamma. In (D), δ = 1 indicates that the known interactions and the unknown interactions are equally important, and the corresponding AUPR value of the model is only 0.42; however, when δ = 2, the model has the maximum AUPR value, which further emphasized that the setting of δ is necessary to improve the performance of the model.

Based on the above discussion, in the following study, we select *η* = 5, µ = 100, λ = 1, γ = 1, *d* = 100, and δ = 2 as parameters of PMDKN.

### Comparison With State-of-the-Art Prediction Methods

In order to evaluate the predictive ability of PMDKN algorithm equitably, we conducted 5-fold cross validation on DATASET 1 and DATASET 2, and compared them with the following methods: SFPEL-LPI (Zhang et al., 2018b), LPLNP (Zhang et al., 2018a), LPBNI (Ge et al., 2016), and LKSNF (Ma et al., 2018b). Since DATASET 1 itself was the benchmark dataset for SFPEL-LPI, LPLNP, and LKSNF, we do not need to re-extract the features. For DATASET 2, we calculated the PCPseDNC and SCPseAAC of lncRNA according to the requirements of SFPEL-LPI, and calculated the PCPseAAC and SCPseAAC of the protein. Since SWSS similarity leads to the reuse of known interaction information, only the Smith Waterman similarity of lncRNA (protein) were calculated. For LPLNP and LKSNF, we calculated the sequence feature and expression profile feature of lncRNA and the CTD of the protein according to their requirements. While LPBNI only uses known lncRNA-protein interactions for prediction, we did not need to extract additional information. According to previous studies, LPLNP, LPBNI, and LKSNF only predicted the unknown interaction of lncRNA-protein, while SFPEL-LPI not only predicted unknown lncRNA-protein interactions, but also predicted new lncRNA and new protein. Therefore, based on DATASET 1 and DATASET 2, we perform *CV_a_* on all models, and *CV_l_* and *CV_p_* on SFPEL-LPI. We performed the crossover experiment using the experimental setup in Section “Experimental Settings” and used the mean of the five-fold crossover experimental results of the five random seeds as the evaluation index of the algorithm, and the parameters of these models were selected using the recommended parameters.

**Table 1** shows the comparison of predictive performance of PMDKN and other state-of-the-art methods for new lncRNA-protein interaction prediction. It can be seen that, no matter in DATASET 1 or DATASET 2, the AUPR, AUC, and F1 values of PMDKN are higher than other models. Specifically, on DATASET 1, as for the most important evaluation index AUPR, PMDKN can reach 0.4959, which increases by 50.46%, 8.37%, 4.31%, and 6.07%, respectively, compared with LPBNI’s 0.3296, LPLNP’s 0.4576, LKSNF’s 0.4754, and SFPEL-LPI’s 0.4675. Regarding the commonly used evaluation index AUC, PMDKN can reach 0.9223, which is higher than 0.8546 of LPBNI, 0.9095 of LPLNP, 0.9150 of LKSNF, and 0.9201 of SFPEL-LPI. The F1 value of PMDKN can reach 0.4814, which is 24.04%, 6.50%, 4% and 3.37%, respectively, compared with 0.3881 for LPBNI, 0.4520 for LPLNP, 0.4629 for LKSNF, and 0.4657 for SFPEL-LPI. In DATASET 2, the AUPR of PMDKN could reach 0.4808, which improved by 40.67%, 2.45%, 6.18%, and 14.07%, respectively, compared with 0.3418 of LPBNI, 0.4693 of LPLNP, 0.4528 of LKSNF, and 0.4215 of SFPEL-LPI. The AUC value of PMDKN can reach 0.9732, higher than 0.9340 of LPBNI, 0.9700 of LPLNP, 0.9710 of LKSNF, and 0.9728 of SFPEL-LPI. The F1 value of PMDKN can reach 0.4761, which is 19.71%, 3.37%, 2.67%, and 7.04%, respectively, compared with 0.3977 for LPBNI, 0.4606 for LPLNP, 0.4637 for LKSNF, and 0.4448 for SFPEL-LPI. These demonstrate that the PMDKN algorithm of this paper has good predictive power for unknown lncRNA-protein interactions.

**Table 1:** Comparison of predicted performance of new lncRNA-protein interactions based on DATASET1 and DATASET2.

DATA	Method	AUPR	AUC	F1 value
DATASET 1	LPBNI	0.3296	0.8546	0.3881
	LPLNP	0.4576	0.9095	0.4520
	LKSNF	0.4754	0.9150	0.4629
	SFPEL-LPI	0.4675	0.9201	0.4657
	PMDKN	**0.4959**	**0.9223**	**0.4814**
DATASET 2	LPBNI	0.3418	0.9340	0.3977
	LPLNP	0.4693	0.9700	0.4606
	LKSNF	0.4528	0.9710	0.4637
	SFPEL-LPI	0.4215	0.9728	0.4448
	PMDKN	**0.4808**	**0.9732**	**0.4761**

In the above table, the best results under the current metric are shown in bold on each data set.

The prediction of new lncRNAs and new proteins are also the important criterion for evaluating the performance of the method. Among the four comparison algorithms above, only SFPEL-LPI performs the prediction of new lncRNA and new protein. Therefore, we only compare the prediction performance of SFPEL-LPI and PMDKN on *CV_l_* and *CV_p_*. As shown in **Table 2**, except for the F1 value of PMDKN on DATASET 2, which is 0.4864, slightly lower than the 0.4892 of SFPEL-LPI, PMDKN was better than SFPEL-LPI for other evaluation indicators, especially for the prediction of new proteins (*CVp*). Specifically, on DATASET 1, the AUPR values of PMDKN for *CV_l_* and *CV_p_* can reach 0.6301 and 0.4918, which is 30.92% and 49.71%, respectively, relative to SFPEL-LPI of 0.4813 and 0.3285. The AUC values of the PMDKN algorithm for *CV_l_* and *CV_p_* can reach 0.8907 and 0.7843, which are 7.52% and 17.66% higher than the 0.8284 and 0.6666 of SFPEL-LPI, respectively. The F1 value of the PMDKN algorithm for *CV_l_* and *CV_p_* can reach 0.6081 and 0.5251, which is 23.32% and 38.95% higher than the 0.4931 and 0.3779 of SFPEL-LPI, respectively. Similarly, in DATASET 2, the AUPR value and AUC value of *CV_l_* of PMDKN were higher than SFPEL-LPI, especially for *CV_p_*, the AUPR value, AUC value, and F1 value of PMDKN could reach 0.4604, 0.9019, and 0.4818, respectively, improving 281.13%, 37.78%, and 148.35% compared with the 0.1208, 0.6546, and 0.1940 of SFPEL-LPI.

**Table 2:** Comparison of predicted performance of new lncRNAs and new proteins based on DATASET1 and DATASET2.

DATA	Method	*CV_l_*			*CV_p_*		
		AUPR	AUC	F1 value	AUPR	AUC	F1 value
DATASET 1	SFPEL-LPI	0.4813	0.8284	0.4931	0.3285	0.6666	0.3779
	PMDKN	**0.6301**	**0.8907**	**0.6081**	**0.4918**	**0.7843**	**0.5251**
DATASET 2	SFPEL-LPI	0.4756	0.9446	**0.4892**	0.1208	0.6546	0.1940
	PMDKN	**0.4794**	**0.9465**	0.4864	**0.4604**	**0.9019**	**0.4818**

In the above table, the best results under the current metric are shown in bold on each data set.

### Comparative Analysis of Model Stability

**Figure 3:**
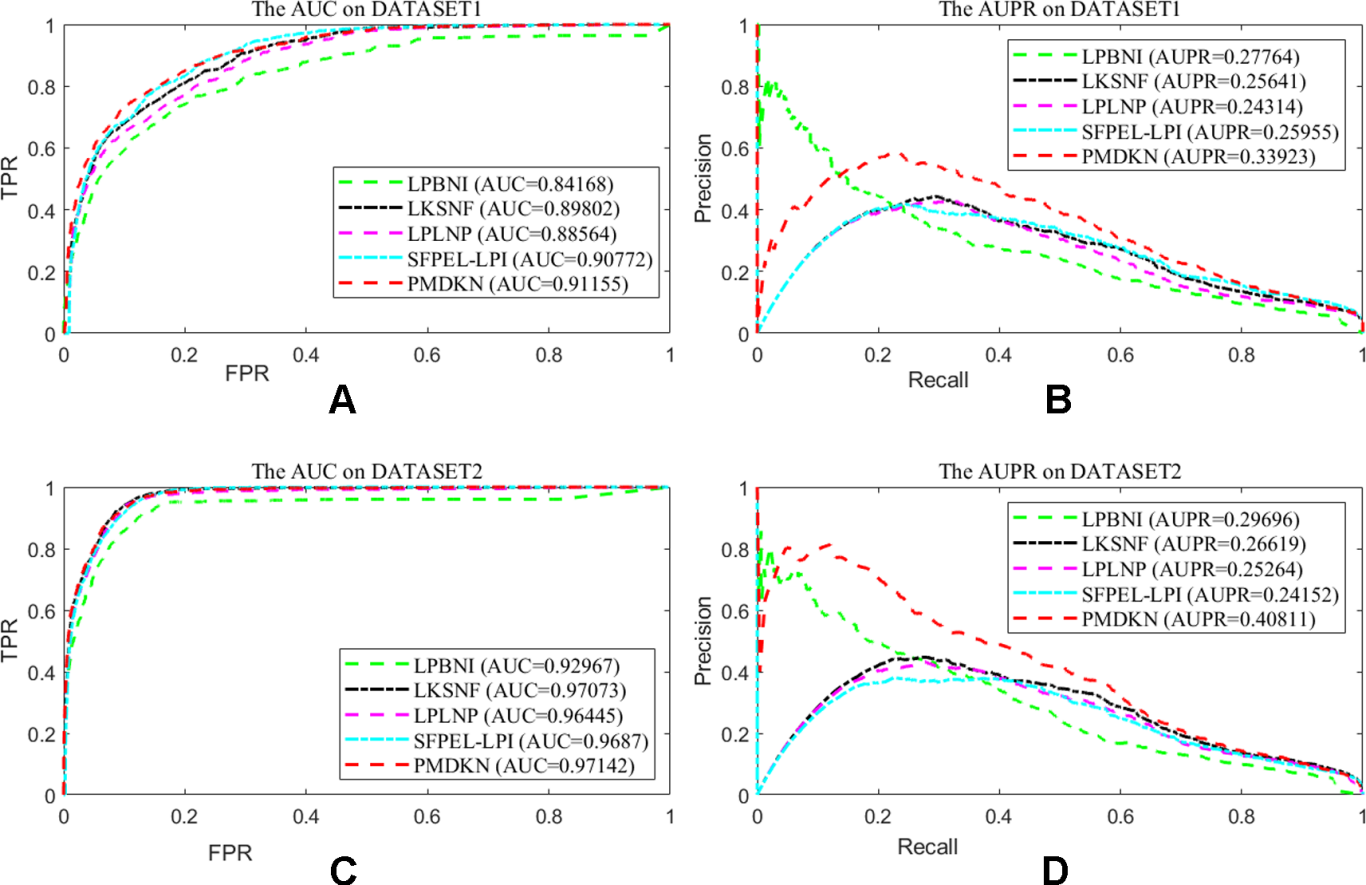
Prediction performance of the model on disturbed data set. Among them, **(A)** shows the ROC curve and AUC value of the five methods after DATASET1 adds noise. **(B)** shows the P-R curve and AUPR values of the five methods after DATASET1 is added with noise. **(C)** shows the ROC curve and AUC value of the five methods after DATASET2 adds noise. **(D)** indicates the P-R curve and AUPR value of the five methods after DATASET2 is added with noise.

Due to technical limitations, some noises may be hidden in the known lncRNA-protein interactions, such as lack of interaction information, unreal interaction information and so on. In order to test the dependence of the prediction performance of the model on the known interactions, according to the method of Zhang et al. (2018b), we randomly deleted some of the known interactions to represent the missing information and randomly added the nonexistent interactions to represent the false interactions, and then studied the change of prediction performance of the model. Since only a few interactions have been detected at present, it indicates that there are still a large number of interactions that have not been discovered. Therefore, we deleted 20% of the known lncRNA-protein interactions and added 5% of the interactions that actually do not exist as noise. At this point, the test set of the model becomes 20% known interactions and all unknown interactions. As shown in **Figure 3**, on the disturbance dataset of DATASET 1, the AUC values of LPBNI, LKSNF, LPLNP, SFPEL-LPI, and PMDKN are 0.8417, 0.8980, 0.8856, 0.9077, and 0.9116, respectively, and the AUPR values are 0.2776, 0.2564, 0.2431, 0.2596, and 0.3392. On the perturbed data set of DATASET 2, the AUC values of LPBNI, LKSNF, LPLNP, SFPEL-LPI, and PMDKN were 0.9297, 0.9707, 0.9646, 0.9687, and 0.9714, respectively, and the AUPR values were 0.2969, 0.2662, 0.2526, 0.2415, and 0.4081, respectively. Comparing the results of **Table 1**, it can be seen that the introduction of partial noise in the perturbed dataset leads to a decrease in the AUPR and AUC values of all prediction models, but PMDKN still achieves satisfactory results and outperforms LPBNI, LKSNF, LPLNP, and SFPEL-LPI.

### Case Study

LncRNA-protein interactions in DATASET 1 and DATASET 2 used in this paper were extracted from Npinter2.0, and the current version of Npinter has been updated to Npinter v3.0 (Hao et al., 2016). Compared with version 2.0(Yuan et al., 2013), Npinter v3.0 contains more lncRNAs, more proteins, and more interactive information. To test the predictive ability of new proteins, we extracted 95 new proteins that did not exist in DATASET 2 from Npinter v3.0, extracted the amino acid sequence and gene ontology annotation of these new proteins, and combined with DATASET2 information to predict the interactions between these new proteins and lncRNAs. For the prediction score of each new protein, we calculated its AUPR and AUC values, and calculated the hit rate of the top 10, 20, 50, and 100 candidate lncRNAs (Nourania et al., 2016). For the new protein *p_i_*, the hit rate hit(*p_i_*) can be expressed as follows:

$$hit\left(p_{i}\right)=\frac{\left||Cand\left(p_{i}\right)\cap Test\left(p_{i}\right)\right|}{\left|\left|Test\left(p_{i}\right)\right|\right|}$$

**Figure 4:**
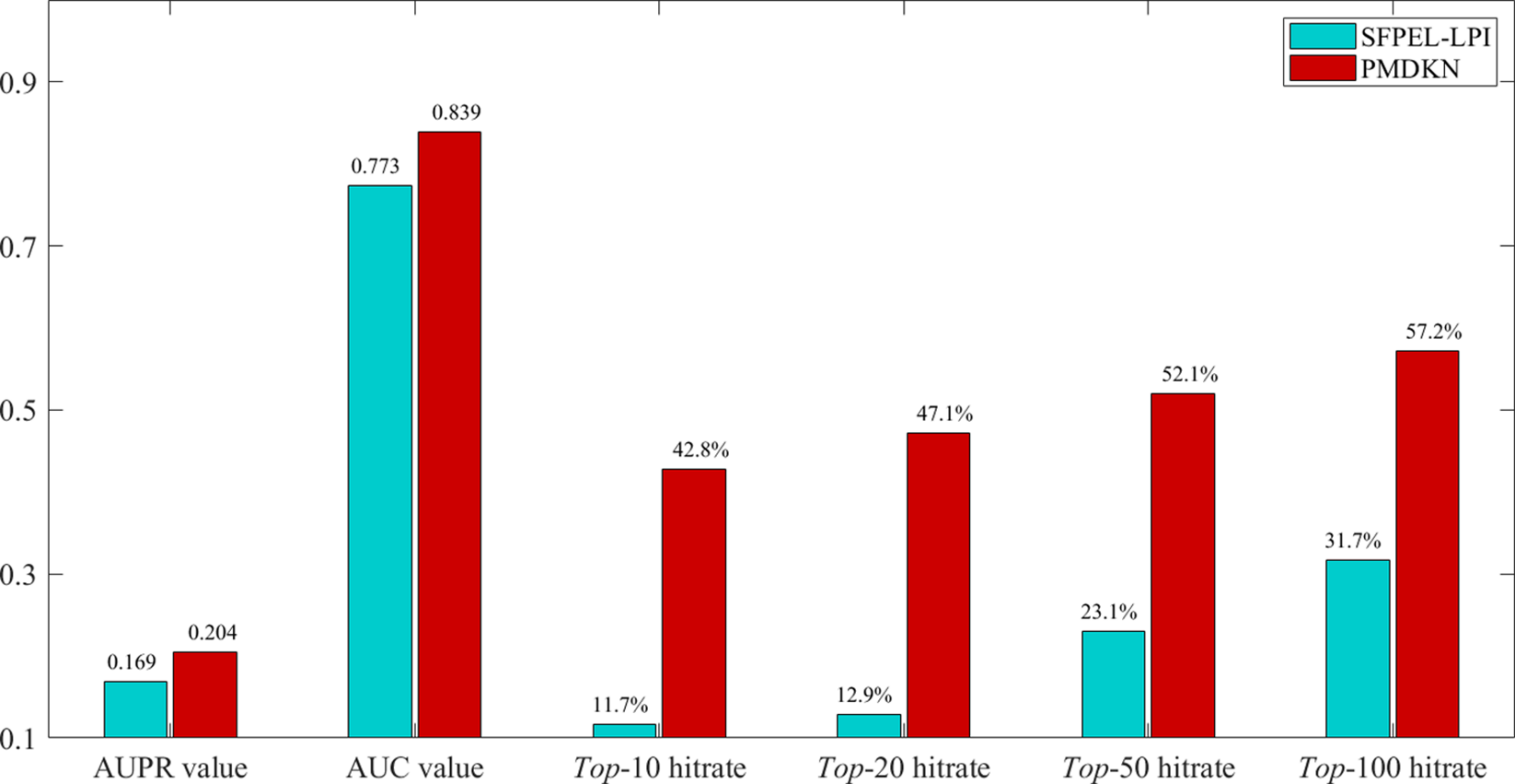
Comparison of SFPEL-LPI and PMDKN prediction results for new proteins. The AUPR and AUC values in the figure represent the average AUPR and average AUC values predicted by PMDKN for 79 proteins, respectively. Top-10 hitrate, Top-20 hitrate, Top-50 hitrate, and Top-100 hitrate represent the mean hit rates of the first 10, 20, 50, and 100 candidate lncRNAs, respectively.

Among them, *Cand (p_i_)* represents the candidate lncRNA set of protein *p_i_*, and in this paper *Cand (p_i_)* represents the *top*-10, *top*-20, *top*-50, *top*-100 candidate lncRNAs sorted according to the predicted score, respectively. *Test (p_i_)* represents the set of lncRNAs for all interactions of protein *p_i_* in Npinter v3.0. |⋅| indicates the number of elements. As SFPEL-LPI can predict new proteins and new lncRNAs, the predicted results of SFPEL-LPI and PMDKN were compared. The predicted scores, actual labels and evaluation indicators of 95 new proteins are shown in **Supplementary Table 1**. The average AUPR value, the average AUC value, the average hit rate of the *top*-10, *top*-20, *top*-50, and *top*-100 predicted by SFPEL-LPI and PMDKN for 95 proteins are shown in **Figure 4**.

As shown in **Figure 4**, for the prediction of new proteins, PDMKN not only has higher AUPR and AUC values than SFPEL-LPI, but also the top 10, 20, 50, 100 hit ratios of candidate lncRNAs are much higher than SFPEL-LPI. Specifically, the average AUPR and AUC values for PMDKN were 0.204 and 0.839, respectively, which were 20.66% and 8.49% higher than 0.169 and 0.773 for SFPEL-LPI, respectively. The hit rates of candidate lncRNAs in the *top*-10, *top*-20, *top*-50 and *top*-100 reached 42.8%, 47.1%, 52.1%, 57.2%, and increased by 266.32%, 264.37%, 125.75%, and 80.68%, respectively, compared with the 11.7%, 12.9%, 23.1%, and 31.7% of SFPEL-LPI, which further demonstrated that PMDKN had strong predictive ability.

## Discussion

In this study, we proposed a new lncRNA-protein interaction prediction model, which not only can predict the unknown interactions between lncRNAs and proteins, but also has strong prediction ability for new lncRNAs and new proteins. To fairly evaluate the predictive performance of the model, we performed three 5-fold cross-validation on the two benchmark datasets, namely, *CV_a_* for the new lncRNA-protein interactions, *CV_i_* for the new lncRNAs, and *CV_p_* for the new proteins. The results show that, on DATASET 1, the AUPR values of PMDKN under the three experimental settings could reach 0.4959 (on *CV_a_*), 0.6301 (on *CV_l_*), and 0.4918(on *CV_p_*) respectively; on DATASET 2, the AUPR values of PMDKN under the three experimental settings can reach 0.4808 (on *CV_a_*), 0.4794 (on *CV_l_*), and 0.4604 (on *CV_p_*) respectively, higher than other state-of-the-art methods. In the case study, 95 new proteins were predicted, and the results showed that for the top-10 candidate lncRNAs, the hit rate of PMDKN algorithm could reach 42.8%, much higher than other method. Therefore, PMDKN can be used as an effective tool for lncRNA-protein interaction prediction.

The good performance of PMDKN may have the following reasons: First, feature extraction and network construction. We extract multiple features to describe lncRNA and protein in all directions and integrate multiple infomation to construct a more accurate lncRNA (protein) similarity network, effectively avoiding the over-fitting problem that may be caused by the information deviation of a single data source. Second, the use of neighborhood information. We modified the initial lncRNA-protein interaction network to overcome the network sparsity problem, and used the adaptive neighborhood completion strategy to eliminate the errors caused by the lack of information in the latent vectors of new lncRNAs (new protein), so as to ensure the predictive ability of new proteins and new lncRNAs. Finally, the construction of the ensemble predictive model. We combine the multiple sequence features of lncRNA (protein) and the integrated similarity networks to construct the predictive model, which distinguishes positive and negative observations by setting important levels and establishes the relationship between features and potential vectors through the projection of the features, so as to improve the accuracy of model prediction.

## Data Availability Statement

The source code and datasets used in the paper can be found in the **Supplementary Files**.

## Author Contributions

YM and XJ designed the projection-based neighborhood non-negative matrix factorization for lncRNA-protein interaction prediction. YM and XJ designed the experiment and wrote the manuscript. TH and XJ supervised and helped conceive the study. All authors read and approved the final manuscript.

## Funding

This research is supported by National Key Research and Development Program of China (2017YFC0909502) and the National Natural Science Foundation of China (61532008 and 61872157).

## Conflict of Interest

The authors declare that the research was conducted in the absence of any commercial or financial relationships that could be construed as a potential conflict of interest.

## Supplementary Material

S1 Table Prediction results of 95 new proteins by SFPEL-LPI and PMDKN.

S1 File Eps format for all pictures in the manuscript.

S2 File The code and data of PMDKN.
